# A more physiological approach to lipid metabolism alterations in cancer: CRC-like organoids assessment

**DOI:** 10.1371/journal.pone.0219944

**Published:** 2019-07-24

**Authors:** Silvia Cruz-Gil, Ruth Sánchez-Martínez, Sonia Wagner-Reguero, Daniel Stange, Sebastian Schölch, Kristin Pape, Ana Ramírez de Molina

**Affiliations:** 1 Molecular Oncology Group/ IMDEA Food Institute, CEI UAM + CSIC, Ctra, De Cantoblanco, Madrid, Spain; 2 Department of Gastrointestinal, Thoracic and Vascular Surgery, Medizinische Fakultät Carl Gustav Carus, Technische Universität Dresden, Dresden, Germany; 3 Department of Surgery, Universitätsmedizin Mannheim, Medical Faculty Mannheim, Heidelberg University, Mannheim, Germany; 4 German Cancer Consortium (DKTK), Dresden, Germany; 5 German Cancer Research Center (DKFZ), Heidelberg, Germany; University of Kentucky, UNITED STATES

## Abstract

Precision medicine might be the response to the recent questioning of the use of metformin as an anticancer drug in colorectal cancer (CRC). Thus, in order to establish properly its benefits, metformin application needs to be assayed on the different progression stages of CRC. In this way, intestinal organoids imply a more physiological tool, representing a new therapeutic opportunity for CRC personalized treatment to assay tumor stage-dependent drugs. The previously reported lipid metabolism-related axis, Acyl-CoA synthetases/ Stearoyl-CoA desaturase (ACSLs/SCD), stimulates colon cancer progression and metformin is able to rescue the invasive and migratory phenotype conferred to cancer cells upon this axis overexpression. Therefore, we checked ACSL/SCD axis status, its regulatory miRNAs and the effect of metformin treatment in intestinal organoids with the most common acquired mutations in a sporadic CRC (CRC-like organoids) as a model for specific and personalized treatment. Despite ACSL4 expression is upregulated progressively in CRC-like organoids, metformin is able to downregulate its expression, especially in the first two stages (I, II). Besides, organoids are clearly more sensitive in the first stage (Apc mutated) to metformin than current chemotherapeutic drugs such as fluorouracil (5-FU). Metformin performs an independent “Warburg effect” blockade to cancer progression and is able to reduce crypt stem cell markers expression such as LGR5+. These results suggest a putative increased efficiency of the use of metformin in early stages of CRC than in advanced disease.

## Introduction

Colorectal cancer (CRC) is the third most common cancer in men (10% of the total), after lung and prostate cancer, and the second in women (9.2% of the total), after breast cancer [1]. Most of the CRC cases are sporadic (70–80%), which consists of the acquisition of somatic mutations and in which there is no family history or genetic predisposition. The remaining cases (20–30%) are those among close relatives, which are divided into inherited or familial CRC [2]. Genetically, sporadic CRC development is due to the accumulation of multiple genetic abnormalities in tumor suppressor genes and oncogenes [3]. Previous research postulated the adenoma-carcinoma transition theory in which specific somatic mutations promoting tumorigenesis are acquired; proposed by Fearon and Vogelstein (named Vogelgram). The Vogelgram proposes that the adenoma-carcinoma sequence model would start with the loss of the *APC* gene, followed by mutations in *KRAS* or *BRAF* genes, mutations or loss of *TP53* gene and of SMAD family member 4 gene (*SMAD4*) [4].

Over the last decade, the interest in metabolic research with respect to cancer has been expansively increased. The first and most characterized tumor metabolism event to be described is the exacerbated glucose uptake and glycolysis utilization; which even in normoxic condition, are not used for maximal ATP generation via mitochondrial respiration. This phenomenon is denoted as the “Warburg effect”.

Even though lipid-associated pathways are functionally dependent on glucose and glutamine catabolic pathways, they are now a well-recognized and frequently described cancer metabolic feature with a key role in tumorigenesis. This is the case for the ACSLs/SCD axis [5], comprised by ACSL1, ACSL4 and SCD, a lipid metabolism-related network described to promote tumorigenesis through an epithelial-mesenchymal transition (EMT) program. Sánchez-Martínez *et al* postulated that ACSL1 and ACSL4 leads to invasion displaying a lower basal respiration and migration accompanied by a more glycolytic phenotype, respectively, to support tumorigenesis. Furthermore, this could be enhanced due to the probable role of SCD in preventing lipotoxic effects that could result from ACSL overexpression. The mesenchymal phenotype produced upon overexpression of these enzymes is reverted through activation of AMPK signaling performed by the well-known anti-diabetic drug, metformin. Though its mechanism of action is not fully understood, metformin has shown a robust anti-proliferative effect on several types of cancer such as colon, pancreatic, breast, ovarian, prostate and lung cancer cells [6]. Furthermore, metformin has been recently associated with improved survival of cancer patients, including CRC, though its use as an antitumoral agent has not been established yet [7].

The ACSLs/SCD axis pro-tumorigenic activity has been also described to be post-transcriptionally regulated by miRNAs. miR-544a, miR-142, and miR-19b-1 have been proposed as major inhibitors of the network. Moreover, miR-19b-1-3p isoform decreased expression is associated with a poorer survival rate in CRC patients, consistently with its target (ACSLs/SCD axis) involvement in patients relapse [8].

To get insight into the metabolic implication on CRC progression with a special focus on the ACSLs/SCD axis and the effect of metformin, more personalized and physiological tools are needed since most of the available data rely on traditional studies using cancer cell lines cultures. In this way, the organoid culture system opens a new methodological door for *ex vivo* studies.

Adult tissue-derived epithelial organoids, also called “mini-guts” [9] are stereotypic tissue-like structures derived from digestive healthy tissues or tumors which mimics *in vitro* the tissue composition and morphology of their *in vivo* counterparts [10]. This methodology was first established in long-term primary culture from mouse small intestinal crypts to generate epithelial organoids with crypt- and villus-like epithelial domains representing both progenitor and differentiated cells [11]. The organoids technology takes advantage of the intestinal epithelium self-renewing capacity. Organoids starts from LGR5 gut epithelial stem cells forming symmetric cyst structures, which finally will form budding structures resembling intestinal crypts. These budding structures are formed by these LGR5 stem cells flanked by differentiated daughter cells [9].

Organoids are currently being employed in CRC studies and chemotherapy assessment [12,13]. Along with intestinal organoids, similar epithelial organoids culture conditions for other mouse and human digestive epithelial tissues have been also adapted [14–17] including tumor-derived organoids from cancer patients. Importantly, organoids grow as pure epithelial cultures without any contamination of vessels, immune cells or non-transformed mesenchymal which leads to an accurate sequencing or expression profiling [10].

In the present manuscript, the antidiabetic drug metformin has been further demonstrated as an advantageous treatment in organoids resembling first stage (I) of a sporadic CRC while it is able to downregulate the stem cell biomarker LGR5 and Wnt target genes expression in all CRC-like organoid stages. Since metformin success was not related to a Warburg-effect weakening, we have linked its effect with oother metabolic pathways such as the lipid metabolism altered axis ACSLs/SCD in CRC.

## Materials and methods

### CRC-like organoids: culture and maintenance

#### Mice

Mutant intestinal murine organoids were obtained from the Universitätsklinikum Carl Gustav Carus, Dresden. All procedures involving animals were conducted strictly in accordance with FELASA regulations and approved by the animal welfare committees of the Technische Universität Dresden and the Landesdirektion Sachsen prior to initiation of the experiments.

Mice with conditional mutations in Apc, Kras, Tp53 and Smad4 were obtained from the NCI Mouse Repository (Apc floxed, p53 floxed) or the Jackson Laboratory (Kras ^G12D^, p53 ^R172H^ and Smad4 floxed) and interbred to obtain compound mutant mice (Table 1). The parental mouse lines were also described in Table 1. The CRC-like organoid model represents the adenoma-carcinoma sequence with the most common acquired mutations in a sporadic CRC [4]: *APC*^fl/fl^, *KRAS*^G12D/WT^, *P53*^R172H/WT^ and Smad4^fl/fl^ (corresponding to stages I to IV) (Table 2).

**Table 1:** Parental mouse lines designation and publication´s PMID of the mutations in the organoids.

Mutation	Designation	PMID
APC floxed	NCI: 01XAA	17002498
KRAS ^G12D^	JAX: 008179	11323676
P53 ^R172H^	JAX: 008652	15607980
P53 floxed	NCI: 01XC2	11694875
Smad4 floxed	JAX: 017462	11857783

**Table 2:** CRC-like organoids with the acquired mutations related to the stage.

Organoid mutation	CRC-like stage
-	WT
APC^fl/fl^	I
APC^fl/fl^, KRAS^G12D/WT^	II
APC^fl/fl^, KRAS^G12D/WT^, P53^fl/R172H^	III
APC^fl/fl^, KRAS^G12D/WT^, P53^fl/R172H^, Smad4^fl/fl^	IV

Murine organoids mutagenesis were conditioned by the Cre/loxP system. Adenoviral infections were performed as explained in [18] to provide active mutations.

#### Crypt isolation and organoids culture

Crypts were isolated from the murine intestine by incubation of 30 min at 4°C in PBS containing 2 mM EDTA as previously reported [11,19]. Isolated crypts were seeded in Matrigel (Corning Matrigel Matrix). The basic culture medium (Advanced Dulbecco’s modified Eagle Medium DMEM/F12 complemented with penicillin/streptomycin, 10 mmol/L HEPES, 1x Glutamax [Gibco], named ADF +++) was supplemented with: 100 ng/ml Noggin (Peprotech), R-spondin (conditioned medium, 10% final volume), 1x B27 (Invitrogen), 1x N2 (Invitrogen), 1,25 mM N-acetylcysteine (Sigma-Aldrich), 100 μg/mL Primocin (InvivoGen) and 50 ng/mL mEGF (Thermofisher). The complete media was named supplemented ADF +++ media. For passaging, organoids were removed from Matrigel and mechanically dissociated with a glass pipette, pelleted and then transferred to fresh Matrigel [11,14,20]. Splitting was performed twice a week in a 1:3 split ratio. Cultures were kept at 37°C, 5% CO2 in humidity.

### Drugs treatment—viability assays

Cell viability was determined by counting and seeding 1000 crypts in 60% of Matrigel in 48-well plates. After 2 days of culture, organoids were exposed 48 hours to 10 mM metformin (Sigma) or 10, 100 or 150 μM 5-FU (Sigma) in supplemented ADF +++ media, as indicated in the figures. At this point, organoids were collected, split and reseeded for recovery experiments over 72 hours in supplemented ADF +++ media.

Upon treatments (48h) or recovery assays (post-72h), organoids were incubated 3 hours with 3-(4,5-dimethyl-thyazol-2-yl)-2,5-diphenyl-tetrazolium (MTT, Sigma). After discarding the media, 20 μl of 2% SDS (Sigma) solution in H2O was added to solubilize Matrigel (2 h, 37 °C). The resultant formazan was dissolved in 100 μl of DMSO for 1 h (37 °C). The absorbance was measured on a microplate reader (Asys UVM 340, Isogen Life Science) at 562 nm.

Vehicle-treated (PBS) control organoids were defined as 100% viable. Data were expressed as the fold change of viable cells from treated organoids compared to the vehicle-treated organoids.

### RNA isolation and RT-QPCR

For RNA isolation, organoids were released from Matrigel with cold Dispase (Corning) and pelleted by centrifugation. The supernatant was removed and pelleted organoids were carefully resuspended in Trizol (Qiagen), and storage at -80°C. RNA was isolated according to the supplier’s protocol (Invitrogen) and the concentration and purity (A260/A280 ratio) were determined by spectrophotometric analysis (NanoDrop 2000 Spectrophotometer ThermoScientific). 20 ng/μl RNA was reverse-transcribed using the High Capacity RNA-to-cDNA kit (ThermoFisher), according to manufacturer’s instructions. Relative gene expression was measured using VeriQuest Fast SYBR Green qPCR Master Mix (2X) (Isogen). Primers used were listed in S1 Table. Regarding miRNAs, their expression was monitored using TaqMan MicroRNA Reverse Transcription Kit (ThermoFisher Scientific) and TaqMan miRNA probes for RT-qPCR (S2 Table). RT-QPCRs were performed on the QuantStudio 12K Flex (Applied Biosystems) and the 2^-ΔΔCt^ method was applied to calculate the relative gene or miRNA expression.

### L-Lactate quantification

Organoids were seeded at a density of 1000 crypts per well in a 48-well plate. After 48 hours, the medium was changed to PBS, 10 mM of metformin or 10 μM of 5-FU in supplemented ADF +++ media overnight at 37°C before quantification. Using Cayman’s Glycolysis cell-based assay (Cayman, Ann Arbor, MI, USA, 600450), extracellular L-Lactate was measured by determining absorbance at 490 nm. L-Lactate measurements (mM) were normalized to total protein concentration (mg) x100.

### Statistical analysis

All statistical analyses were performed using the Graph Pad Prism software (Ver. 7.03) (GraphPad Software, San Diego, CA, USA). The differences between treatments were tested by one-way analysis of variances (ANOVA), followed by post-hoc multiple comparisons to identify which particular treatments were significantly different. In this case, Bonferroni method was applied to avoid type-I error inflated. The significance level was set to 0.05. In the graphs, p-values are represented as ns, *P ≥* 0.05; *, *P* ≤ 0.05; **, *P* ≤ 0.01; ***, *P* ≤ 0.001; ****, *P* ≤ 0.0001. All values are reported as mean ± S.D.

## Results

### ACSL4 is overexpressed throughout CRC-like organoids stages

**Fig 1:**
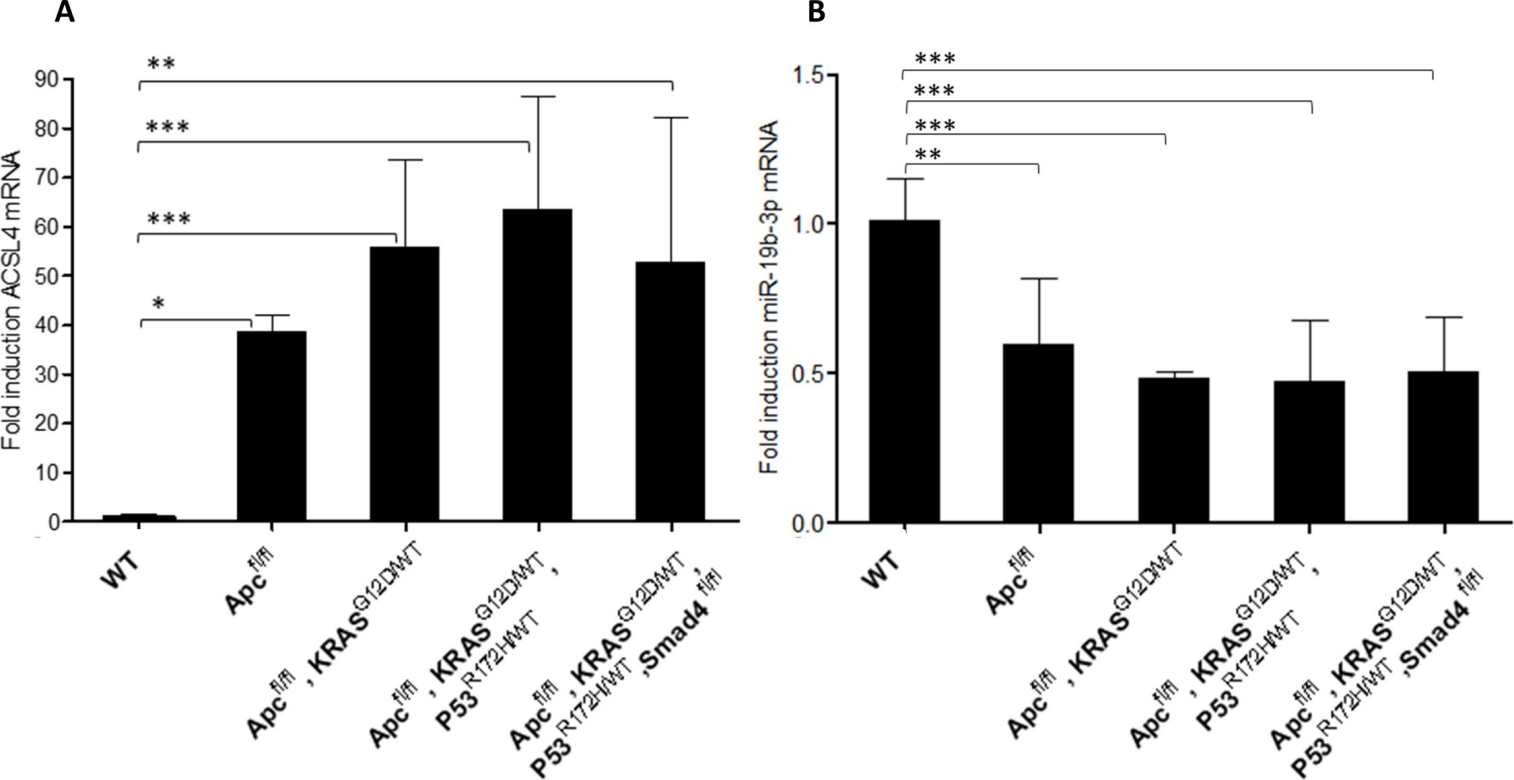
ACSL4 is overexpressed throughout CRC-like organoids stages while miR-19b-1-3p preserves its protective role. A) RT-QPCR analysis showing ACSL4 mRNA expression levels throughout CRC-like organoids stages. B) RT-QPCR analysis showing miR-19b-1-3p mRNA expression levels throughout CRC-like organoids stages. Results represent the fold-change mean ±SD (*n* = 4) in plots A, (*n* = 3) in plots B. (ns, *P* > 0.05; *, *P* ≤ 0.05; **, *P* ≤ 0.01; ***, *P* ≤ 0.001).

ACSL4 has been previously reported overexpressed in malignant tumors, and together with ACSL1 and SCD form a metabolic axis involved in CRC progression. ACSL1, ACSL4, and SCD mRNA expression were measured in CRC-like organoids. ACSL4 mRNA expression was very significantly augmented in more aggressive stages compared to WT (Fig 1A). Conversely, ACSL1 and SCD levels were maintained or increased from the third stage henceforth, respectively (S1 Fig).

Interestingly, organoids in more advanced stages (III and IV) presented a genetic misbalance in ACSL4 expression (Fig 1A) with huge differences in their fold inductions ranges in the same stage, though with a similar tendency.

### MiR-19b-1-3p keeps its protective role in CRC-like organoids

Previous results from our group pointed toward a correlation between miR-19b-1-3p lower expression and a poorer prognosis in CRC patients; very likely through its involvement in cell invasion and lipid metabolism regulation [8]. This might have a putative clinical interest due to miRNAs potential to be assayed in plasma as non-invasive biomarkers. MiRNAs expression was assayed in three different RNA extractions over time. In the case of CRC-like organoids, this tendency was maintained and miR-19b-1-3p expression was decreased in a stage-dependent manner (Fig 1B).

Together with miR-19b-1-3p, miR-142 (3p and 5p isoforms) and miR-544a (without murine isoform) were also involved in targeting ACSL/SCD axis [8]. Hence, the previously mentioned miRs plus miR-19b-1-5p isoform was measured though no statistically significant differences were found in its expression (S2 Fig)

### Metformin decreases CRC-like organoids viability to the same extent as current chemotherapy without significant effects on WT organoids

**Fig 2:**
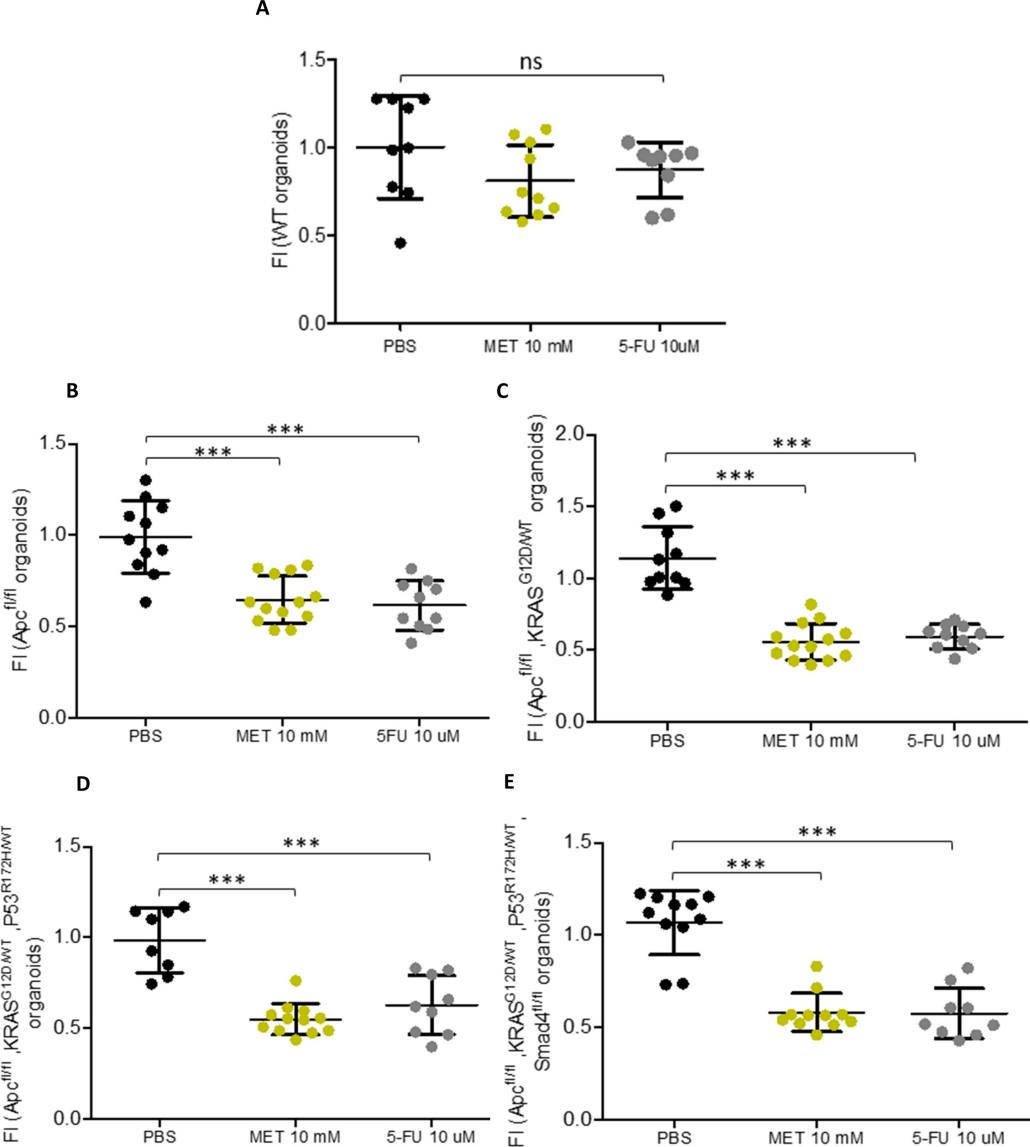
Metformin decreases CRC-like organoids viability to the same extent as current chemotherapy without significant effects on WT organoids. MTT cell viability assays upon 48 hours treatments with metformin or 5-FU in CRC-like organoids representative stages (A) WT organoids; (B) *APC*^fl/fl^ organoids resembling stage I; (C) *APC*^fl/fl^, *KRAS*^G12D/WT^ organoids resembling stage II; (D) *APC*^fl/fl^, *KRAS*^G12D/WT^, *P53*^R172H/WT^ organoids resembling stage III; (E) *APC*^fl/fl^, *KRAS*^G12D/WT^, *P53*^R172H/WT^, Smad4^fl/fl^ organoids resembling stage IV. Data are represented by the fold-change mean ±SD (*n* = 3) in all the plots (ns, *P* > 0.05; *, *P* ≤ 0.05; **, *P* ≤ 0.01; ***, *P* ≤ 0.001).

Since metformin, an AMPK activator, was able to rescue the epithelial phenotype from the EMT process caused by the overexpression of ACSL/SCD in CRC cells [5]; we assayed this drug effect through the different stages in CRC-like organoids progression. CRC-like organoids were treated with PBS (vehicle control), 10 mM of metformin or with the commonly used chemotherapeutic agent 5-FU. The organoids viability was examined by MTT assays 48 hours upon treatment. None of the drugs affected significantly the viability of WT organoids (Fig 2A), while they were able to cause a decrease of about 50% in the viability of all mutated organoids corresponding to the most aggressive phenotypes (Fig 2B–2E). 5-FU higher concentrations (100 μM and 150 μM) showed the same effects as the lower concentration (10 μM) in mutated organoids, while showing stronger effects on WT ones (S3A–S3E Fig).

### Metformin treatment viability recovery is significantly lower compared to 5-FU in first stage organoids while WT organoids present an opposite behavior

**Fig 3:**
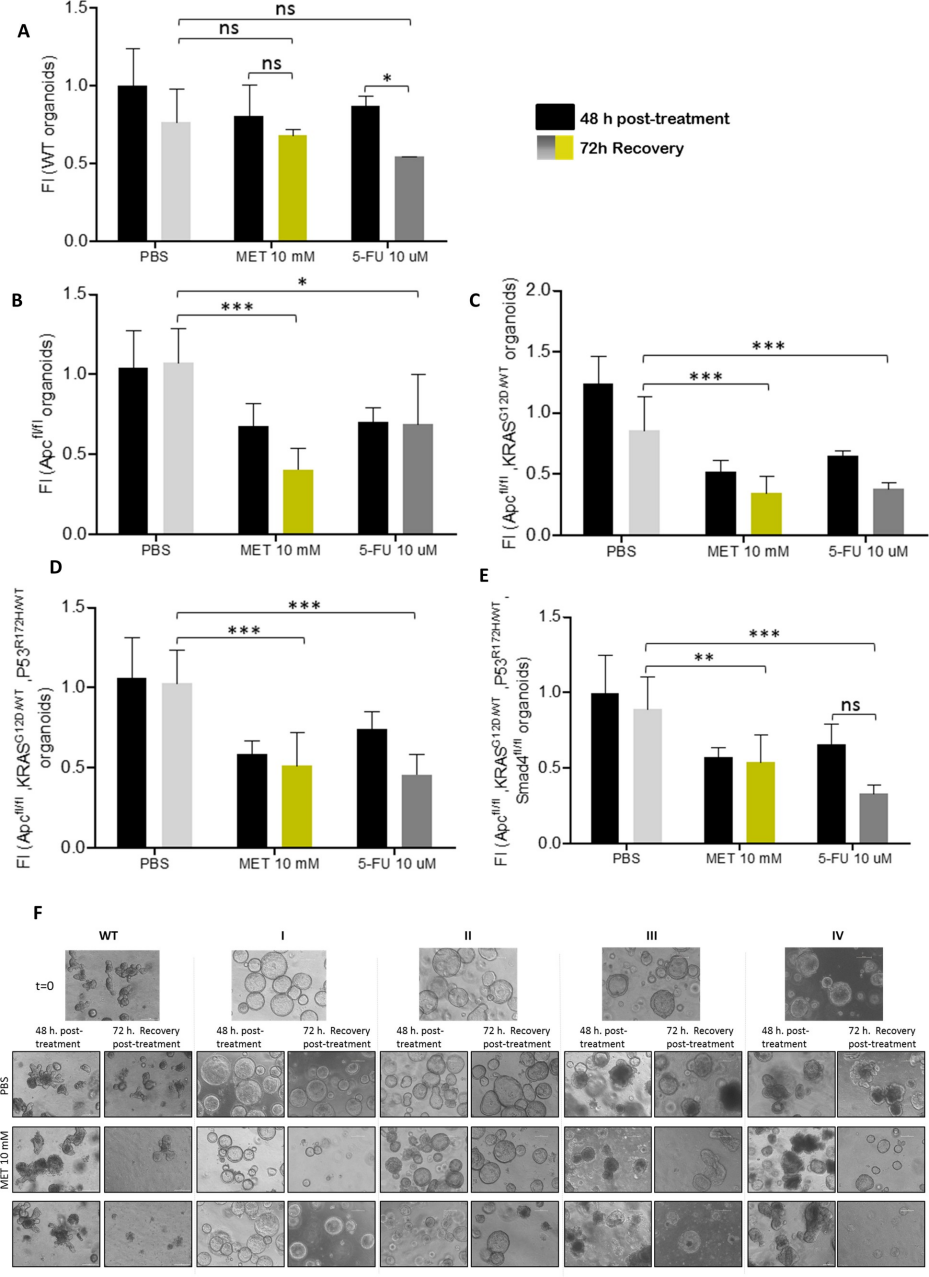
Metformin treatment viability recovery is significantly lower compared to 5-FU in first stage organoids while WT organoids present an opposite behavior. MTT cell viability assays upon 48 hours treatments (black bars) and upon extra 72h post-treatment recovery with PBS (light grey bars), 10 mM metformin (yellow bars) or 10 μM 5-FU (dark grey bars) in CRC-like organoids representative stages: (A) WT organoids; (B) *APC*^fl/fl^ organoids resembling stage I; (C) *APC*^fl/fl^, *KRAS*^G12D/WT^ organoids resembling stage II; (D) *APC*^fl/fl^, *KRAS*^G12D/WT^, *P53*^R172H/WT^ organoids resembling stage III; (E) *APC*^fl/fl^, *KRAS*^G12D/WT^, *P53*^R172H/WT^, Smad4^fl/fl^ organoids resembling stage IV. Data are represented by the fold-change mean ±SD (*n* = 3) in all the plots. (ns, *P*> 0.05; *, *P* ≤ 0.05; **, *P* ≤ 0.01; ***, *P* ≤ 0.001). (F) Organoids pictures with PBS, metformin or 5-FU, upon 48 hours treatments and upon extra 72h post-treatment recovery as indicated. Pictures were captured using the x10 objective, in bright field. Leica microscope (Leica Microsystems).

To further check the treatment scope, and to analyze not only the effect but also the potential reversibility of the treatment in normal and tumoral cells in different stages, organoids viability was assayed upon 48 hours treatment (PBS, metformin or 5-FU) plus the subsequent recovery of 72 additional hours in their growing media. In this case, WT organoids showed differential recovery sensitivity to the treatment. Metformin treated and recovered WT organoids presented almost similar measurements to PBS control recovered organoids. Nonetheless, 5-FU treated WT organoids recoveries are noteworthy more sensitive and upon 72h recovery time, their viability was quite significantly reduced (*p-value*: **) (Fig 3A). In Apc mutated organoids the recovery was very significantly lower upon metformin treatment (*p-value*: ***) than 5-FU (*p-value*: *), compared to PBS recovery control; making these Apc mutated organoids the most responsive to the metformin treatment compared to 5-FU (Fig 3B). Regarding organoids corresponding to stages II-III (Fig 3C and 3D), both treatments presented almost similar recovery effects, while in stage IV organoids, 5-FU presented a stronger effect shown by the lower recovery of these organoids (Fig 3E). Again 5-FU higher concentrations treatment (100 μM and 150 μM) had nearly the same recovery effects than the lower concentration (10 μM) in CRC-like organoids (S4A–S4E Fig).

By way of clarification, all CRC-like organoids presented Apc gene loss since it is the first gene in the adenocarcinoma sequence. Apc completed deletion provokes a hyperactive Wnt signaling. This aberration makes an organoids phenotype switch, losing their crypt-like structure and adopting a cystic morphology [17,21]. On the contrary, WT organoids maintain this crypt-like structure as could be observed in Fig 3F. Since this complex structure requires more time for the organoid to grow, pictures from the treatment recoveries show WT organoids with smaller sizes, even with the initial cyst-like structures. Besides that, stage I (Apc^fl/fl^) organoids recovered upon metformin treatment showed a significantly reduced size compared with the control and the 5-FU treated ones (S5 Fig). In accordance with viability assays results, this effect is lost in further stages; where metformin is less effective than the ones treated with 5-FU.

### Metformin action is stronger on ACSL4 and SCD overexpressing first stages organoids (I & II)

Since stage I organoids seemed to present a differential sensitivity to metformin compared to other stages, together with differential expression of ACSL4, we aimed to analyze the possible link between metformin effect on ACSL/SCD axis in intestinal organoids.

**Fig 4:**
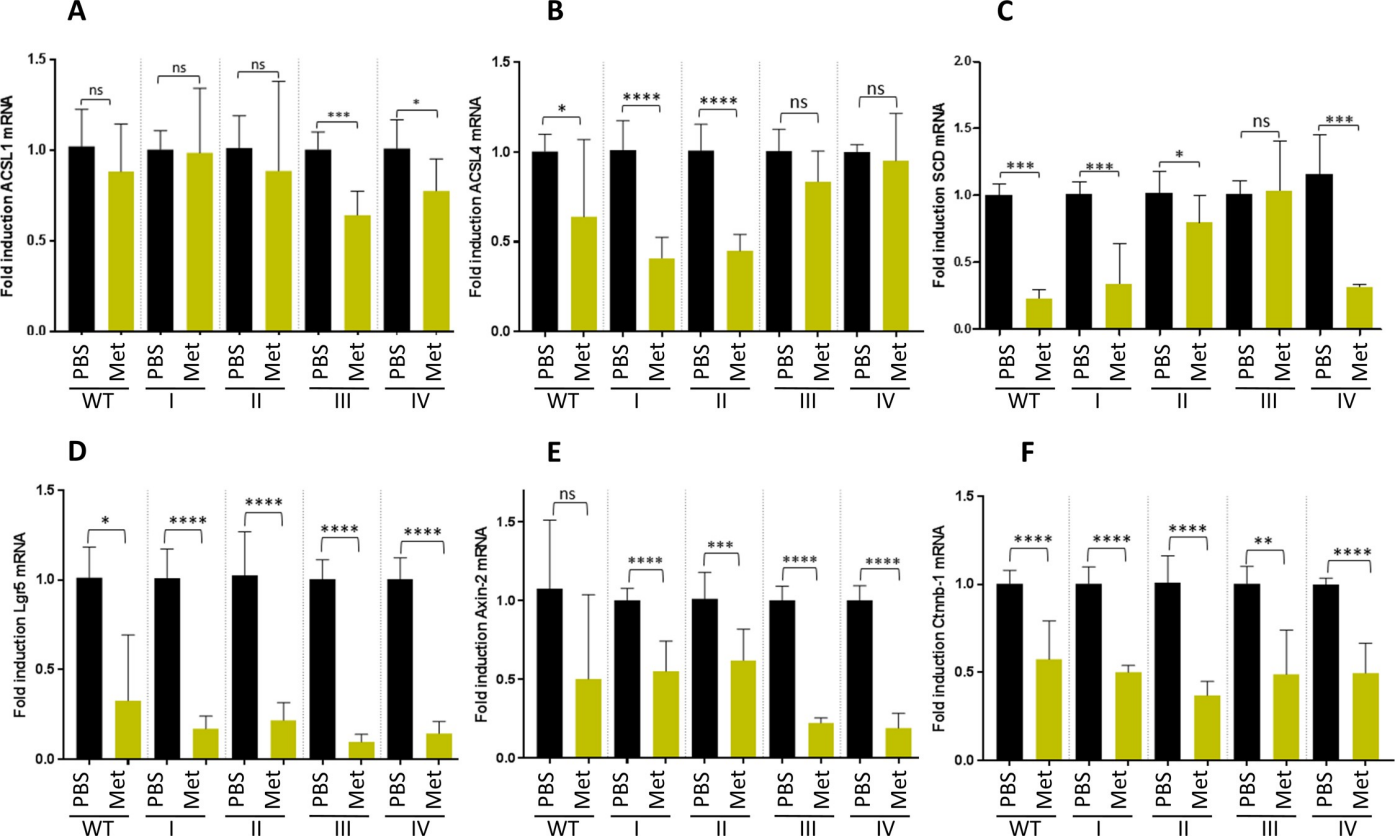
Metformin action is stronger on ACSL4 and SCD overexpressing first stages organoids (I & II) and downregulates the intestinal stem cell biomarker LGR5 and Wnt target genes expression in all organoid stages. mRNA expression levels of the genes comprising the ACSL/SCD axis, ACSL1 (A), ACSL4 (B) and SCD (C), by RT-QPCR; and expression levels of different stem cell markers, LGR5 (D), Axin-2 (E) and Ctnnb-1 (F) by RT-QPCR upon PBS (black bars) and 10 mM metformin (yellow bars). Data are represented by the fold-change mean to each PBS control ±SD (*n* = 3). (ns, *P*> 0.05; *, *P* ≤ 0.05; **, *P* ≤ 0.01; ***, *P* ≤ 0.001; ****, *P* ≤ 0.0001).

To this aim, ACSL4 expression was measured, as well as the other enzymes conforming the metabolic network (ACSL1 and SCD) upon 10 mM metformin treatment. ACSL4 mRNA expression was strongly reduced by this drug compared to their PBS-treated controls in stage I and II organoids. By contrast, stage III and IV presented no significance in their ACSL4 mRNA reduction upon metformin treatment. WT organoids also presented a slight reduction of ACSL4 mRNA upon metformin treatment (Fig 4B). In addition, SCD expression levels were clearly decreased by metformin in WT and stage I organoids, while a less marked tendency was found for stage II, III and IV organoids stage (Fig 4C). ACSL1 mRNA analysis showed less significant and more variable results (Fig 4A) upon metformin treatment.

The expression levels of these enzymes were also measured upon 10 μM 5-FU treatment. This drug was able to significantly downregulate ACSL4 and SCD mRNA in most of the stages, though no differences were showed between the effects in initial and later stages like metformin treatment (S6A–S6C Fig).

### Metformin, but not 5-FU, downregulates the intestinal stem cell biomarker LGR5 and Wnt target genes expression in all CRC-like organoids stages

To further assay whether metformin treatment was targeting the organoids crypts stem cell marker, LGR5; we analyzed its expression together with two other Wnt target genes, Axin2 and Ctnnb-1. Importantly, LGR5 expression was significantly diminished in the whole CRC-like organoids series upon metformin treatment (Fig 4D) as well as Axin-2 (Fig 4E) and Ctnnb-1 (Fig 4E) mRNAs. Surprisingly, these patterns were not maintained when organoids were treated with 10 μM 5-FU (S6D–S6F Fig).

### Metformin action in CRC-like organoids is not related to a Warburg-effect impairment

**Fig 5:**
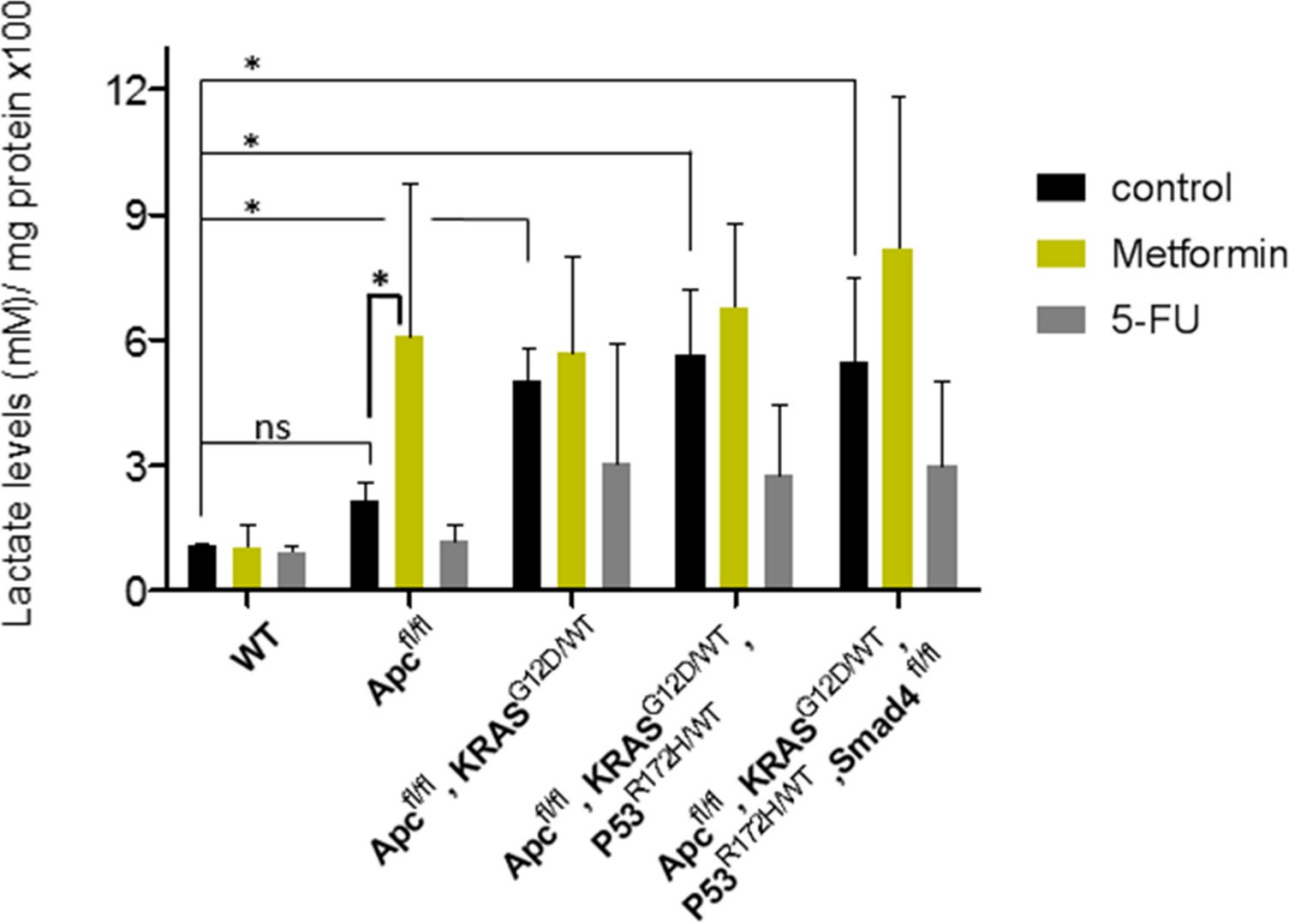
Metformin action in CRC organoids is not related to a Warburg-effect impairment. Bars represent the extracellular L-lactate production upon overnight PBS treatment (black bars), 10 mM metformin treatment (yellow bars) and 10 μM 5-FU treatment (grey bars) using the Cayman’s Glycolysis cell-based assay. L-lactate production measurement is normalized by total protein content (x100). Data are represented by the fold-change mean ±SD (*n* = 3) in all the plots. (ns, *P*> 0.05; *, *P* ≤ 0.05).

The avidity to perform glycolysis even in the presence of oxygen, known as the Warburg effect, is one of the hallmarks of tumors. For this reason, we measured the levels of L-lactate, the end product of glycolysis. CRC-like organoids presented increased glycolysis compared to WT organoids, reflecting an increasing Warburg effect throughout the stages, as expected. Even though 5-FU treatment caused a slight decrease in the glycolytic performance of the mutant organoids (Fig 5), metformin treatment caused an opposite effect, increasing the glycolytic capacity in all stages, especially in stage I, the most sensitive to the drug. Thus, it seems that metformin effect on CRC-like organoids viability relies on mechanisms other than preventing pro-tumorigenic Warburg effect, likely through the regulation of lipid metabolism.

## Discussion

Organoids represent a suitable tool to study lipid metabolism [22] and previous studies employing intestinal organoids have linked the critical role of fatty acid metabolism to the intestinal epithelial integrity *in vivo* [23]. Therefore, we propose this system to get insight into cancer progression mechanisms in regards to fatty acid metabolism and therefore, to assay ACSL/SCD protumorigenic axis action in CRC.

We showed that ACSL4 augmented while miR-19b-1-3p diminished its expression, both progressively, in murine CRC-like organoids. Metformin action compared to the chemotherapeutic agent 5-FU, in terms of viability reduction, was similar; although no significant reduction was found in WT organoids viability with any treatment. Stage I organoids were the most susceptible to metformin action after 72 h recovery time compared to 5-FU; while further stages (III, IV) presented similar or stronger sensitivity to 5-FU, including WT organoids. Besides, metformin was able to reduce the intestinal crypt stem cell marker LGR5 in all the stages, together with two other Wnt downstream targets, Ctnnb-1 and Axin-2. Finally, we showed that even though CRC-like organoids series presented a growing Warburg effect through the stages consistent with increased L-lactate levels; metformin action on CRC organoids viability was not related to ablation of the Warburg metabolism, suggesting other metabolic targets.

The individual role of ACSL isoform 1 [24,25] and 4 [24,26] as well as SCD [27–31] has been extensively reported in CRC. Surprisingly, while ACSL4 mRNA levels are clearly increased through the stages in this organoids model, this was not the case for ACSL1; and SCD was only overexpressed in advanced stages. These results differ from previously reported ones using human CRC cells which can be due to differential expression in murine tissues compared to human 2D cultures [5,8]. Nevertheless, the use of murine organoids allow their genetic engineering and to accurately control the mutations for better mechanistic characterization, rather than patient tumor-derived biopsies with the high variability that each tumor represent. Thus, the CRC model employed in the present manuscript mimics a sporadic colorectal tumor with the common mutations acquired during the progression of this cancer. In this murine model, the overexpression of ACSL4 is preserved (Fig 1A), probably indicating a predominant role of this ACSL/SCD component in cancer progression aspects. The ACSL4 mRNA huge range of expression considering the most mutated stages (Fig 1A) could be explained since stages III and IV in real tumors present an uncontrolled genetic variability with the accumulation of other undetermined mutations. Organoids would be mimicking these uncontrolled stages, compared to the homogeneity presented in 2D cultures. Conversely, ACSL1 static role (S1A Fig) could be due to a lesser implication of this enzyme in tumor development in this system which can be also explained by the fact that the rodent protein is one residue longer (699 amino acids) than the human protein (698 amino acids), making it necessary to study the extent of this dissimilarity. For its part, SCD overexpression has been mainly reported in mesenchymal tissues [32,33], rather than epithelial ones, which are the only scaffold for organoids giving a reason for the distinctive results found in these epithelial systems among the first stages (S1B Fig).

Regarding miRs expression, miR-19b-1-3p kept its tumor-suppressor role in murine CRC-like organoids, also reported as a good prognosis miRNA, able to target the protumorigenic ACSLs/SCD axis [8]. The immature isoform of miR-19b-1-3p, miR-19b, and other members of the miR-17-92 cluster, where this miRNA is involved, regulate the self-renewal ability of gastric cancer stem cells [34]. The miR-17-92 cluster role is controversial and dependent on the cancer type [35,36]. However, it is interesting the reported role of this miRNA in digestive cancer stem cells, and its role in CRC stem cells may be a potential line of research henceforth. In line with our results, miR-19b was also reported to downregulate suppressor of cytokine signaling 3 (SOC3), modulating chemokine production in intestinal epithelial cells and thereby avoiding intestinal inflammation in Crohn’s disease, which may ultimately prevent the derived disease, CRC [37].

Since metformin was able to revert the ACSL/SCD EMT phenotype, we tried to gain insight on this process using organoid cultures resembling the different stages of a CRC progression. Metformin treatment seems to be more efficient than 5-FU only during first tumor stages (I), making organoids recovery harder compared to the ones treated with 5-FU. We proposed that metformin therapies could be an appealing alternative in those cases when the tumor is detected in very early stages rather than 5-FU treatments. However, some studies testing metformin treatment in CRC patients points to stage III to be the most likable to present an effect [38]. Still, this study reported a low number of candidates in stage I, probably not enough representative. Taking these factors into account, the main challenge lies in the fact that CRC is hard to be detected on its very early stages, known as one of the most silent and deadly cancers.

As well, metformin therapies have been proposed alone or in combination with other drugs, in CRC. For example, metformin has been recently combined with aspirin to treat middle stages in non-diabetic CRC patients (II and III stages) [39]. Furthermore, it exists a Phase 2 Trial for the study of metformin and 5-FU combination in metastatic CRC [40], concluded with longstanding cancer control. An older report also claimed the benefits of this combination, but they also reported that metformin alone has antineoplastic activity *per se* in colon cancer cells, and enhanced the activity of 5-FU, oxaliplatin and irinotecan in cells previously treated [41].

Previous reports hypothesized that the inhibition of mitochondrial complex I was the main mechanism of action for metformin. However, recent studies suggest that cancer progression is compromised upon metformin treatment by decreasing the TCA cycle´s anaplerosis. Metformin decreases the flow of glucose- and glutamine-derived metabolic intermediates into the TCA cycle, decreasing the citrate output of the mitochondria and leading to a reduction of acetyl-CoA (Ac-CoA) and oxaloacetate (OAA) in the cytoplasm and therefore a reduction in *de novo* FA synthesis [42]. This way, metformin could be targeting lipid metabolism through ACSL/SCD axis. ACSL4 downregulation in the presence of metformin is clearly evident and the results are larger significant in first stages (I, II) (Fig 4B). Probably, the intermediate overexpression of ACSL4 in the first stage (Apc ^fl/fl^) (Fig 1A) increases the sensitivity to metformin action (Fig 4B); while in more advanced stages, the overexpression is so high that metformin action could be less effective. This would be the same for SCD, which showed no overexpression in the first stages and enhanced overexpression in III and IV stages, and it is again significantly reduced upon metformin exposure in the first stages (I & II) (Fig 4C), when the overexpression is not out of control. In the case of ACSL1 (Fig 4A) since we have not found overexpression throughout the stages it seems metformin is only affecting to advanced stages, complementing ACSL4 downregulation in first stages for the purpose of blocking tumorigenesis. On the other hand, it has been reported that variations in the types and amounts of fatty acids are able to modify intracellular ACSLs expression [43], thus, these conditions could be also affecting ACSLs/SCD enzymes expression. Besides, the network connection could make them present coordinated effects upon metformin treatment, reducing their expression due to the lack of substrate (fatty acids). In fact, metformin was previously reported to downregulate ACSL expression, lowering fatty acid synthesis and normalizing lipid profile in diabetic rats [44]; as well as limiting its products, 18-carbon chain length fatty acids, in skeletal muscle insulin resistant rats [45]; suggesting in this case that metformin is increasing FAs mitochondrial channeling due to the reduction of CPT1 inhibition by malonyl-CoA and therefore decreasing 18-carbon acyl-chain-derived bioactive lipids in the cytoplasm [45]. This role of metformin could be added to the aforementioned, detoxifying ACSLs probable over activity.

Metformin seemed also to target cancer stem cells in different cancer types [46]. However, we have described for the first time the LGR5 downregulation in CRC-like organoids upon metformin treatment; consistent with previous reports using 2D CRC cell cultures [47]. LGR5 was diminished in the whole CRC-like organoids series to minimum levels, an indicative that metformin action is affecting the stem cells of the crypt, responsible for the progression of the organoids lineage. Curiously, metformin treated organoids do not present apoptosis or even necrosis, but they kept at a minimum size compared to other treatments (S5 Fig), where the organoids layer disappeared and the cells appeared apoptotic in the lumen (S7 Fig), showing that cell membrane biogenesis is somehow blocked, mostly built by *de novo* lipogenesis routes.

Finally, metformin treated CRC organoids exhibit a greater compensatory increase in aerobic glycolysis. Since ATP levels are diminished due to complex I inhibition, the metabolic sensor AMPK is activated, inhibiting mTOR and proliferative events; and promoting glycolysis as an alternative ATP source [48]. We found that even though the CRC-organoids series presented increasing glycolysis throughout the stages, (Fig 5); metformin was able to increase even more this glycolytic phenotype, especially in stage I organoids, coincident with the higher sensitivity to the drug in this phase. These results pointed towards metformin targeting a different metabolic route other than Warburg effect to perform its effect on CRC organoids viability.

Even though the Warburg effect is a priority for current drugs, the evidence grows pointing to other metabolic pathways to be targeted for cancer progression ablation. CRC is a leading cause of death in the developed world, though yet simplistic preclinical models that mimic the usual stages of CRC progression are lacking [13]. In this way, organoids further analysis need to be included as a tool of choice for stage-dependent drugs screening.

## Conclusions

### General conclusion

1 Organoids display a precise platform to assay **tumor stage-dependent drugs** being suitable for **personalized medicine**, constituting an invaluable tool due to their relatively low costs, animal saving suffering and their ease and legibility of being genetically manipulated.

### Metformin-related conclusions

2 **Metformin** treatment is further proved as an efficient drug in CRC:
  - It is able to decrease CRC-like organoids viability at the same rate as current chemotherapy (5-FU) but it does not affect WT organoids.
  - Metformin treatment recovery is significantly inferior compared to 5-FU in **first stages organoids**, but with a greater recovery in WT organoids; becoming an appealing chemotherapy drug in first tumor phases.
  - Metformin downregulates the stem cell biomarker LGR5 and Wnt target genes expression in all CRC-like organoid stages, reaffirming its potential use in intestinal cancers.
  - Metformin action in CRC organoids is not related to a Warburg-effect impairment, presuming that other metabolisms rather than Warburg should be targeted to complete the cancer progression obstruction.

### ACSL/SCD-related conclusions

3 **ACSL4** is progressively overexpressed throughout CRC-like organoids stages; while **miR-19b-1-3p** preserves its protective role, reflecting ACSLs/SCD axis action on CRC progression. Besides, metformin action is stronger on ACSL4 and SCD-overexpressing first stages organoids, agreeing with metformin greater action on this stage.

## Supporting information

S1 Fig ACSL1 and SCD mRNA expression throughout CRC-like organoids stages. RT-QPCR analysis showing ACSL1 (A) and SCD (B) mRNA expression levels throughout CRC- like organoids stages. Results represent the fold-change mean ±SD (*n* = 3) (ns, *P* > 0.05; *, *P* ≤ 0.05; **, *P* ≤ 0.01; ***, *P* ≤ 0.001).
(TIF)

S2 Fig ACSL/SCD regulatory miRNAs expression in CRC-like organoids. RT-QPCR analysis showing mRNA expression levels throughout CRC-like organoids stages of different ACSL/SCD regulatory miRNAS: miR-19b-1-5p (A), miR-142-3p (B), miR-142-5p (C). Results represent the fold-change mean ±SD (*n* = 3). (ns, *P*> 0.05).
(TIF)

S3 Fig Metformin and 5-FU effect in CRC-like organoids. MTT cell viability assays upon 48 hours treatments with PBS (black bars), 10 mM metformin (yellow bars) or 10, 100 and 150 uM 5-FU (grey bars) in the different CRC-like organoids representative stages: WT organoids (A); *APC*fl/fl organoids resembling stage I (B); *APC*fl/fl, *KRAS*G12D/WT organoids resembling stage II (C); *APC*fl/fl, *KRAS*G12D/WT, *P53*R172H/WT organoids resembling stage III (D); *APC*fl/fl, *KRAS*G12D/WT, *P53*R172H/WT, Smad4fl/fl organoids resembling stage IV (E). Data are represented by the fold-change mean ±SD (*n* = 3) in all the plots. (ns, *P* > 0.05; *, *P* ≤ 0.05; **, *P* ≤ 0.01; ***, *P* ≤ 0.001).
(TIF)

S4 Fig Metformin and 5-FU recovery effect in CRC-like organoids. MTT cell viability assays upon 48 hours treatments (black bars) and upon extra 72h post-treatment recovery with PBS (light grey bars), 10 mM metformin (yellow bars) or 10, 100 and 150 uM 5-FU (dark grey bars) in the different CRC-like organoids representative stages: WT organoids (A); *APC*fl/fl organoids resembling stage I (B); *APC*fl/fl, *KRAS*G12D/WT organoids resembling stage II (C); *APC*fl/fl, *KRAS*G12D/WT, *P53*fl/R172H organoids resembling stage III (D); *APC*fl/fl, *KRAS*G12D/WT, *P53*fl/R172H,Smad4fl/fl organoids resembling stage IV (E). Data are represented by the fold-change mean ±SD (*n* = 3) in all the plots. (ns, *P* > 0.05; *, *P* ≤ 0.05; **, *P* ≤ 0.01; ***, *P* ≤ 0.001).
(TIF)

S5 Fig Metformin treatment viability recovery generates organoids with a smaller diameter compared to 5-FU in first stage organoids. Organoids diameter measured with Image J 1.48 program from pictures of the MTT cell viability assays upon 48 hours treatments (black bars) and upon extra 72h post-treatment recovery with PBS (light grey bars), 10 mM metformin (yellow bars) or 10 μM 5-FU (dark grey bars) in *APC*^fl/fl^ organoids resembling stage I. Data are represented by the fold-change mean ±SD (*n* = 3 experiments/3 images analyzed per condition) (ns, *P*> 0.05; *, *P* ≤ 0.05; **, *P* ≤ 0.01). Significance between groups was determined by unpaired *t*-test analyses. Pictures were captured using the x10 objective, in bright field. Leica microscope (Leica Microsystems).
(TIF)

S6 Fig ACSL/SCD axis and stem cell markers expression (Lgr5, Axin-2 and Ctnnb-1) upon metformin and 5-FU treatment. Expression levels of enzymes related to the ACSL/SCD axis, ACSL1 (A), ACSL4 (B) and SCD (C) by RT-QPCR; and expression levels of different stem cell markers, Lgr5 (D), Axin-2 (E) and Ctnnb-1 (F) by RT-QPCR; upon PBS (black bars), 10 mM metformin (yellow bars) or 10, 100 and 150 uM 5-FU (grey bars). Data are represented by the fold-change mean ±SD (*n* = 3). (ns, *P*> 0.05; *, *P* ≤ 0.05; **, *P* ≤ 0.01; ***, *P* ≤ 0.001).
(TIF)

S7 Fig Comparative organoids morphology between metformin and other oncologic treatments. Organoids (stage I and III) representative pictures with DMSO, metformin and other metabolic drugs against CRC progression, upon 48 hours treatments plus upon extra 72h post-treatment recovery. Pictures were captured using the × 10 objective, in bright field. Leica microscope (Leica Microsystems).
(TIF)

S1 Table Primers´ sequences (Invivogen) used for quantitative real-time PCR. (DOCX)

S2 Table Probes from TaqMan MicroRNA Assays (ThermoFisher) used for quantitative real-time PCR. (DOCX)

## Abbreviations

ACSL1: Acyl-CoA synthetase 1
ACSL4: Acyl-CoA synthetase 4
CRC: Colorectal cancer
EMT: Epithelial-mesenchymal transition
5-FU: 5-Fluorouracil
MiRNAs/ miRs: MicroRNAs
MTT: 3-(4, 5-dimethylthiazol-2-yl)-2, 5-diphenyltetrazolium bromide
OAA: Oxaloacetate
SCD: Stearoyl-CoA desaturase
TCA: Tricarboxylic Acid cycle
2D: 2-dimensional
3D: 3-dimensional

## References

[1] Ferlay J, Soerjomataram I, Dikshit R, Eser S, Mathers C, Rebelo M, et al Cancer incidence and mortality worldwide: sources, methods and major patterns in GLOBOCAN 2012. Int J Cancer. 2015;136: E359–386. 10.1002/ijc.29210 25220842

[2] Müller MF, Ibrahim AEK, Arends MJ. Molecular pathological classification of colorectal cancer. Virchows Arch. 2016;469: 125–134. 10.1007/s00428-016-1956-3 27325016 PMC4978761

[3] Yamagishi H., Kuroda H., Imai Y., & Hiraishi H. (2016). Molecular pathogenesis of sporadic colorectal cancers. Chinese Journal of Cancer, 35, 4 10.1186/s40880-015-0066-y 26738600 PMC4704376

[4] Fearon ER, Vogelstein B. A genetic model for colorectal tumorigenesis. Cell. 1990;61: 759–767. 10.1016/0092-8674(90)90186-i 2188735

[5] Sánchez-Martínez R, Cruz-Gil S, Gómez de Cedrón M, Álvarez-Fernández M, Vargas T, Molina S, et al A link between lipid metabolism and epithelial-mesenchymal transition provides a target for colon cancer therapy. Oncotarget. 2015;6: 38719–38736. 10.18632/oncotarget.5340 26451612 PMC4770732

[6] Nasri H, Rafieian-Kopaei M. Metformin: Current knowledge. J Res Med Sci Off J Isfahan Univ Med Sci. 2014;19: 658–664. PMC4214027 25364368

[7] Baglia ML, Cui Y, Zheng T, Yang G, Li H, You M, et al Diabetes Medication Use in Association with Survival among Patients of Breast, Colorectal, Lung, or Gastric Cancer. Cancer Res Treat Off J Korean Cancer Assoc. 2018; 10.4143/crt.2017.591 29986576 PMC6473299

[8] Cruz-Gil S, Sanchez-Martinez R, Gomez de Cedron M, Martin-Hernandez R, Vargas T, Molina S, et al Targeting the lipid metabolic axisACSL/SCDin colorectal cancer progression by therapeutic miRNAs: miR-19b-1 role. J Lipid Res. 2018;59: 14–24. 10.1194/jlr.M076752 29074607 PMC5748493

[9] Sato T, Clevers H. SnapShot: Growing Organoids from Stem Cells. Cell. 2015;161: 1700–1700.e1. 10.1016/j.cell.2015.06.028 26091044

[10] Werner K, Weitz J, Stange DE. Organoids as Model Systems for Gastrointestinal Diseases: Tissue Engineering Meets Genetic Engineering. Curr Pathobiol Rep. 2016;4: 1–9. 10.1007/s40139-016-0100-z

[11] Sato T, Vries RG, Snippert HJ, van de Wetering M, Barker N, Stange DE, et al Single Lgr5 stem cells build crypt-villus structures in vitro without a mesenchymal niche. Nature. 2009;459: 262–265. 10.1038/nature07935 19329995

[12] Golovko D, Kedrin D, Yilmaz ÖH, Roper J. Colorectal cancer models for novel drug discovery. Expert Opin Drug Discov. 2015;10: 1217–1229. 10.1517/17460441.2015.1079618 26295972 PMC4872297

[13] O’Rourke KP, Loizou E, Livshits G, Schatoff EM, Baslan T, Manchado E, et al Transplantation of engineered organoids enables rapid generation of metastatic mouse models of colorectal cancer. Nat Biotechnol. 2017;35: 577–582. 10.1038/nbt.3837 28459450 PMC5462850

[14] Barker N, Huch M, Kujala P, van de Wetering M, Snippert HJ, van Es JH, et al Lgr5(+ve) stem cells drive self-renewal in the stomach and build long-lived gastric units in vitro. Cell Stem Cell. 2010;6: 25–36. 10.1016/j.stem.2009.11.013 20085740

[15] Boj SF, Hwang C-I, Baker LA, Chio IIC, Engle DD, Corbo V, et al Organoid models of human and mouse ductal pancreatic cancer. Cell. 2015;160: 324–338. 10.1016/j.cell.2014.12.021 25557080 PMC4334572

[16] Huch M, Dorrell C, Boj SF, van Es JH, Li VSW, van de Wetering M, et al In vitro expansion of single Lgr5+ liver stem cells induced by Wnt-driven regeneration. Nature. 2013;494: 247–250. 10.1038/nature11826 23354049 PMC3634804

[17] Sato T, Stange DE, Ferrante M, Vries RGJ, Van Es JH, Van den Brink S, et al Long-term expansion of epithelial organoids from human colon, adenoma, adenocarcinoma, and Barrett’s epithelium. Gastroenterology. 2011;141: 1762–1772. 10.1053/j.gastro.2011.07.050 21889923

[18] Seidlitz T, Merker SR, Rothe A, Zakrzewski F, von Neubeck C, Grützmann K, et al Human gastric cancer modelling using organoids. Gut. 2018; 10.1136/gutjnl-2017-314549 29703791 PMC6352409

[19] Barker N, Huch M, Kujala P, van de Wetering M, Snippert HJ, van Es JH, et al Lgr5(+ve) stem cells drive self-renewal in the stomach and build long-lived gastric units in vitro. Cell Stem Cell. 2010;6: 25–36. 10.1016/j.stem.2009.11.013 20085740

[20] Farin HF, Van Es JH, Clevers H. Redundant sources of Wnt regulate intestinal stem cells and promote formation of Paneth cells. Gastroenterology. 2012;143: 1518–1529.e7. 10.1053/j.gastro.2012.08.031 22922422

[21] Andersson-Rolf A, Mustata RC, Merenda A, Kim J, Perera S, Grego T, et al One-step generation of conditional and reversible gene knockouts. Nat Methods. 2017;14: 287–289. 10.1038/nmeth.4156 28135257 PMC5777571

[22] Bijsmans ITGW, Milona A, Ijssennagger N, Willemsen ECL, Ramos Pittol JM, Jonker JW, et al Characterization of stem cell-derived liver and intestinal organoids as a model system to study nuclear receptor biology. Biochim Biophys Acta. 2017;1863: 687–700. 10.1016/j.bbadis.2016.12.004 27956139

[23] Ciorba MA. Scap and the intestinal epithelial stem cell niche: new insights from lipid biology. J Lipid Res. 2015;56: 1381–1382. 10.1194/jlr.C061309 26063459 PMC4513980

[24] Chen W-C, Wang C-Y, Hung Y-H, Weng T-Y, Yen M-C, Lai M-D. Systematic Analysis of Gene Expression Alterations and Clinical Outcomes for Long-Chain Acyl-Coenzyme A Synthetase Family in Cancer. PLoS ONE. 2016;11 10.1371/journal.pone.0155660 27171439 PMC4865206

[25] Heimerl S, Moehle C, Zahn A, Boettcher A, Stremmel W, Langmann T, et al Alterations in intestinal fatty acid metabolism in inflammatory bowel disease. Biochim Biophys Acta. 2006;1762: 341–350. 10.1016/j.bbadis.2005.12.006 16439103

[26] Cao Y, Dave KB, Doan TP, Prescott SM. Fatty acid CoA ligase 4 is up-regulated in colon adenocarcinoma. Cancer Res. 2001;61: 8429–8434. 11731423

[27] Roongta UV, Pabalan JG, Wang X, Ryseck R-P, Fargnoli J, Henley BJ, et al Cancer cell dependence on unsaturated fatty acids implicates stearoyl-CoA desaturase as a target for cancer therapy. Mol Cancer Res MCR. 2011;9: 1551–1561. 10.1158/1541-7786.MCR-11-0126 21954435

[28] Igal RA. Stearoyl CoA desaturase-1: New insights into a central regulator of cancer metabolism. Biochim Biophys Acta. 2016;1861: 1865–1880. 10.1016/j.bbalip.2016.09.009 27639967

[29] Chen L, Ren J, Yang L, Li Y, Fu J, Li Y, et al Stearoyl-CoA desaturase-1 mediated cell apoptosis in colorectal cancer by promoting ceramide synthesis. Sci Rep. 2016;6: 19665 10.1038/srep19665 26813308 PMC4728559

[30] Ran H, Zhu Y, Deng R, Zhang Q, Liu X, Feng M, et al Stearoyl-CoA desaturase-1 promotes colorectal cancer metastasis in response to glucose by suppressing PTEN. J Exp Clin Cancer Res CR. 2018;37: 54 10.1186/s13046-018-0711-9 29530061 PMC5848567

[31] Qiu Y, Cai G, Zhou B, Li D, Zhao A, Xie G, et al A distinct metabolic signature of human colorectal cancer with prognostic potential. Clin Cancer Res Off J Am Assoc Cancer Res. 2014;20: 2136–2146. 10.1158/1078-0432.CCR-13-1939 24526730 PMC5902798

[32] Lu Y, Zhou Z, Tao J, Dou B, Gao M, Liu Y. Overexpression of stearoyl-CoA desaturase 1 in bone marrow mesenchymal stem cells enhance the expression of induced endothelial cells. Lipids Health Dis. 2014;13: 53 10.1186/1476-511X-13-53 24650127 PMC3974181

[33] Tao J, Shi J, Lu Y, Dou B, Zhou Z, Gao M, et al Overexpression of stearoyl-CoA desaturase 1 in bone-marrow mesenchymal stem cells increases osteogenesis. Panminerva Med. 2013;55: 283–289. 24088802

[34] Wu Q, Yang Z, Wang F, Hu S, Yang L, Shi Y, et al MiR-19b/20a/92a regulates the self-renewal and proliferation of gastric cancer stem cells. J Cell Sci. 2013;126: 4220–4229. 10.1242/jcs.127944 23868977

[35] Xiang J, Wu J. Feud or Friend? The Role of the miR-17-92 Cluster in Tumorigenesis. Curr Genomics. 2010;11: 129–135. 10.2174/138920210790886853 20885820 PMC2874222

[36] Zhang K, Zhang L, Zhang M, Zhang Y, Fan D, Jiang J, et al Prognostic value of high-expression of miR-17-92 cluster in various tumors: evidence from a meta-analysis. Sci Rep. 2017;7: 8375 10.1038/s41598-017-08349-4 28827775 PMC5567103

[37] Cheng X, Zhang X, Su J, Zhang Y, Zhou W, Zhou J, et al miR-19b downregulates intestinal SOCS3 to reduce intestinal inflammation in Crohn’s disease. Sci Rep. 2015;5 10.1038/srep10397 25997679 PMC4441154

[38] Lee JH, Kim TI, Jeon SM, Hong SP, Cheon JH, Kim WH. The effects of metformin on the survival of colorectal cancer patients with diabetes mellitus. Int J Cancer. 2012;131: 752–759. 10.1002/ijc.26421 21913184

[39] De Monte A, Brunetti D, Cattin L, Lavanda F, Naibo E, Malagoli M, et al Metformin and aspirin treatment could lead to an improved survival rate for Type 2 diabetic patients with stage II and III colorectal adenocarcinoma relative to non-diabetic patients. Mol Clin Oncol. 2018;8: 504–512. 10.3892/mco.2018.1554 29456855 PMC5795555

[40] Miranda VC, Braghiroli MI, Faria LD, Bariani G, Alex A, Bezerra Neto JE, et al Phase 2 Trial of Metformin Combined With 5-Fluorouracil in Patients With Refractory Metastatic Colorectal Cancer. Clin Colorectal Cancer. 2016;15: 321–328.e1. 10.1016/j.clcc.2016.04.011 27262895

[41] Effect of metformin alone and in combination with 5-fluorouracil (5FU), oxaliplatin (O) and irinotecan (I) on human colon cancer cell lines.: Journal of Clinical Oncology: Vol 29, No 15_suppl [Internet]. [cited 26 Jul 2018]. Available: http://ascopubs.org/doi/abs/10.1200/jco.2011.29.15_suppl.e13041

[42] Griss T, Vincent EE, Egnatchik R, Chen J, Ma EH, Faubert B, et al Metformin Antagonizes Cancer Cell Proliferation by Suppressing Mitochondrial-Dependent Biosynthesis. PLoS Biol. 2015;13: e1002309 10.1371/journal.pbio.1002309 26625127 PMC4666657

[43] Yan S, Yang X-F, Liu H-L, Fu N, Ouyang Y, Qing K. Long-chain acyl-CoA synthetase in fatty acid metabolism involved in liver and other diseases: an update. World J Gastroenterol. 2015;21: 3492–3498. 10.3748/wjg.v21.i12.3492 25834313 PMC4375570

[44] Ghadge A, Harsulkar A, Karandikar M, Pandit V, Kuvalekar A. Comparative anti-inflammatory and lipid-normalizing effects of metformin and omega-3 fatty acids through modulation of transcription factors in diabetic rats. Genes Nutr. 2016;11 10.1186/s12263-016-0518-4 27551311 PMC4968436

[45] Zabielski P, Chacinska M, Charkiewicz K, Baranowski M, Gorski J, Blachnio-Zabielska AU. Effect of metformin on bioactive lipid metabolism in insulin-resistant muscle. J Endocrinol. 2017;233: 329–340. 10.1530/JOE-16-0381 28522731

[46] Metformin and Cancer Stem Cells: Old Drug, New Targets | Cancer Prevention Research [Internet]. [cited 30 Jul 2018]. Available: http://cancerpreventionresearch.aacrjournals.org/content/5/3/351 10.1158/1940-6207.CAPR-12-0026 22389436

[47] Mogavero A, Maiorana MV, Zanutto S, Varinelli L, Bozzi F, Belfiore A, et al Metformin transiently inhibits colorectal cancer cell proliferation as a result of either AMPK activation or increased ROS production. Sci Rep. 2017;7: 15992 10.1038/s41598-017-16149-z 29167573 PMC5700100

[48] Andrzejewski S, Gravel S-P, Pollak M, St-Pierre J. Metformin directly acts on mitochondria to alter cellular bioenergetics. Cancer Metab. 2014;2: 12 10.1186/2049-3002-2-12 25184038 PMC4147388

