# Correction: A more physiological approach to lipid metabolism alterations in cancer: CRC-like organoids assessment

**DOI:** 10.1371/journal.pone.0222819

**Published:** 2019-09-16

**Authors:** Silvia Cruz-Gil, Ruth Sánchez-Martínez, Sonia Wagner-Reguero, Daniel Stange, Sebastian Schölch, Kristin Pape, Ana Ramírez de Molina

There are errors in the Funding statement. The correct Funding statement is as follows: This work was supported by Spanish Ministry of Science (Plan Nacional I+D+i AGL2016-76736-C3), Regional Government of Community of Madrid (S2018/BAA-4343), Ramón Areces Foundation and EU Structural Funds. Boehringer Ingelheim Fonds granted SCG for a short stage. The funders had no role in study design, data collection and analysis, decision to publish, or preparation of the manuscript.
