# Supplementary figures and images for: A more physiological approach to lipid metabolism alterations in cancer: CRC-like organoids assessment
Source: PLoS One. 2019 Jul 24;14(7):e0219944. doi: 10.1371/journal.pone.0219944 (PMC6655698; doi:10.1371/journal.pone.0219944)

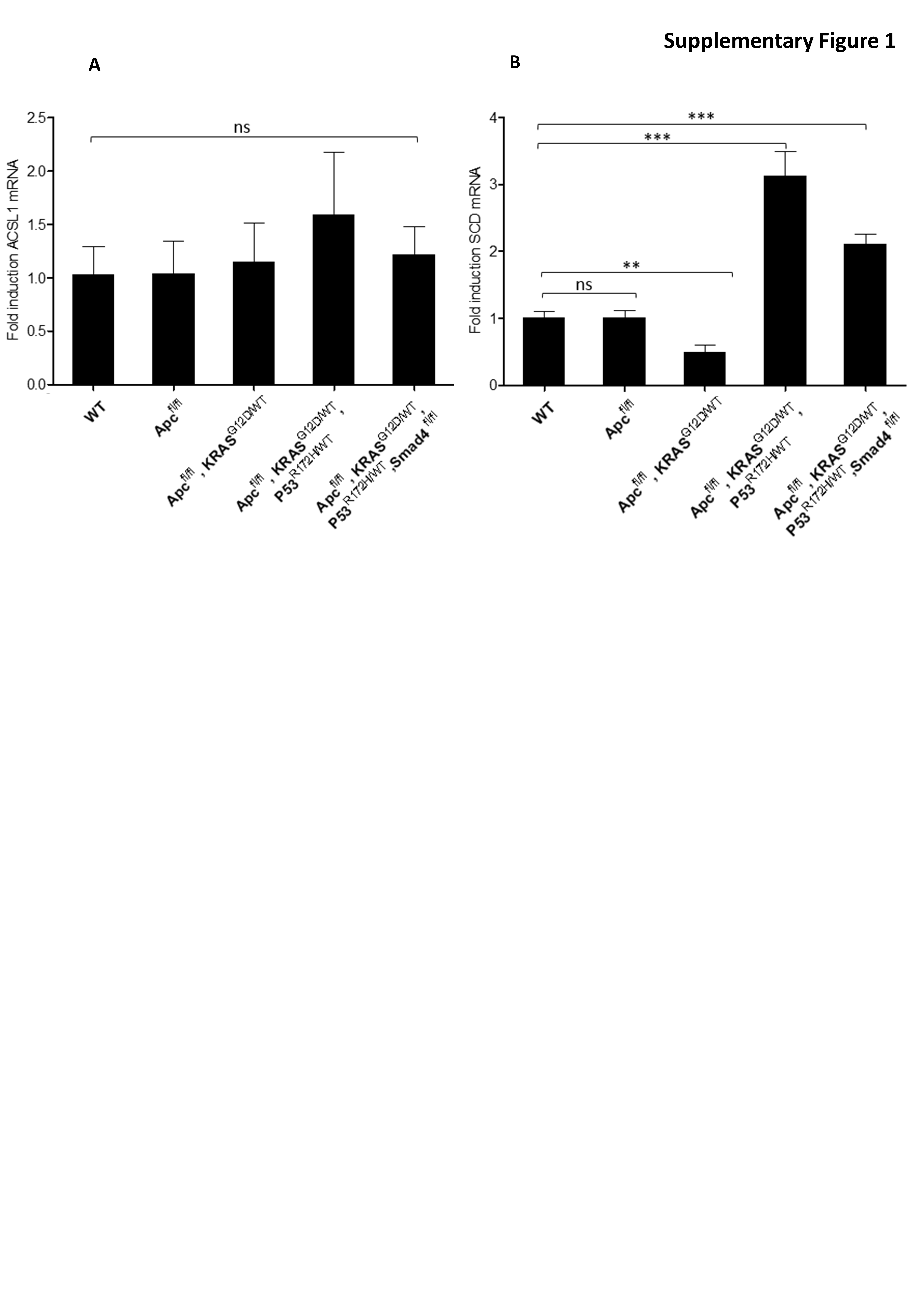

Supplement: S1 Fig — RT-QPCR analysis showing ACSL1 (A) and SCD (B) mRNA expression levels throughout CRC- like organoids stages. Results represent the fold-change mean ±SD (n = 3) (ns, P > 0.05; *, P ≤ 0.05; **, P ≤ 0.01; ***, P ≤ 0.001). (TIF) [file pone.0219944.s001.TIF]

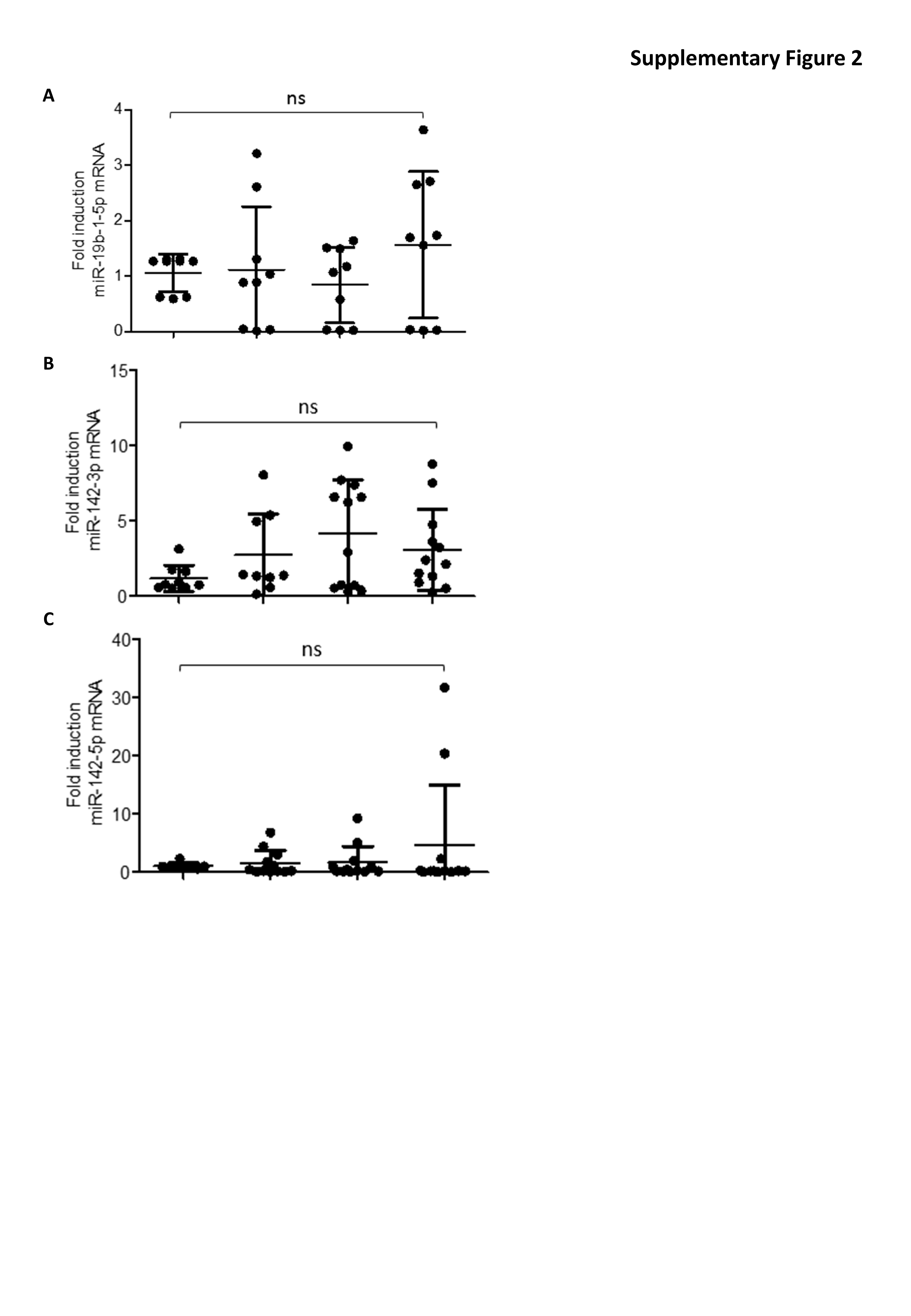

Supplement: S2 Fig — RT-QPCR analysis showing mRNA expression levels throughout CRC-like organoids stages of different ACSL/SCD regulatory miRNAS: miR-19b-1-5p (A), miR-142-3p (B), miR-142-5p (C). Results represent the fold-change mean ±SD (n = 3). (ns, P> 0.05). (TIF) [file pone.0219944.s002.TIF]

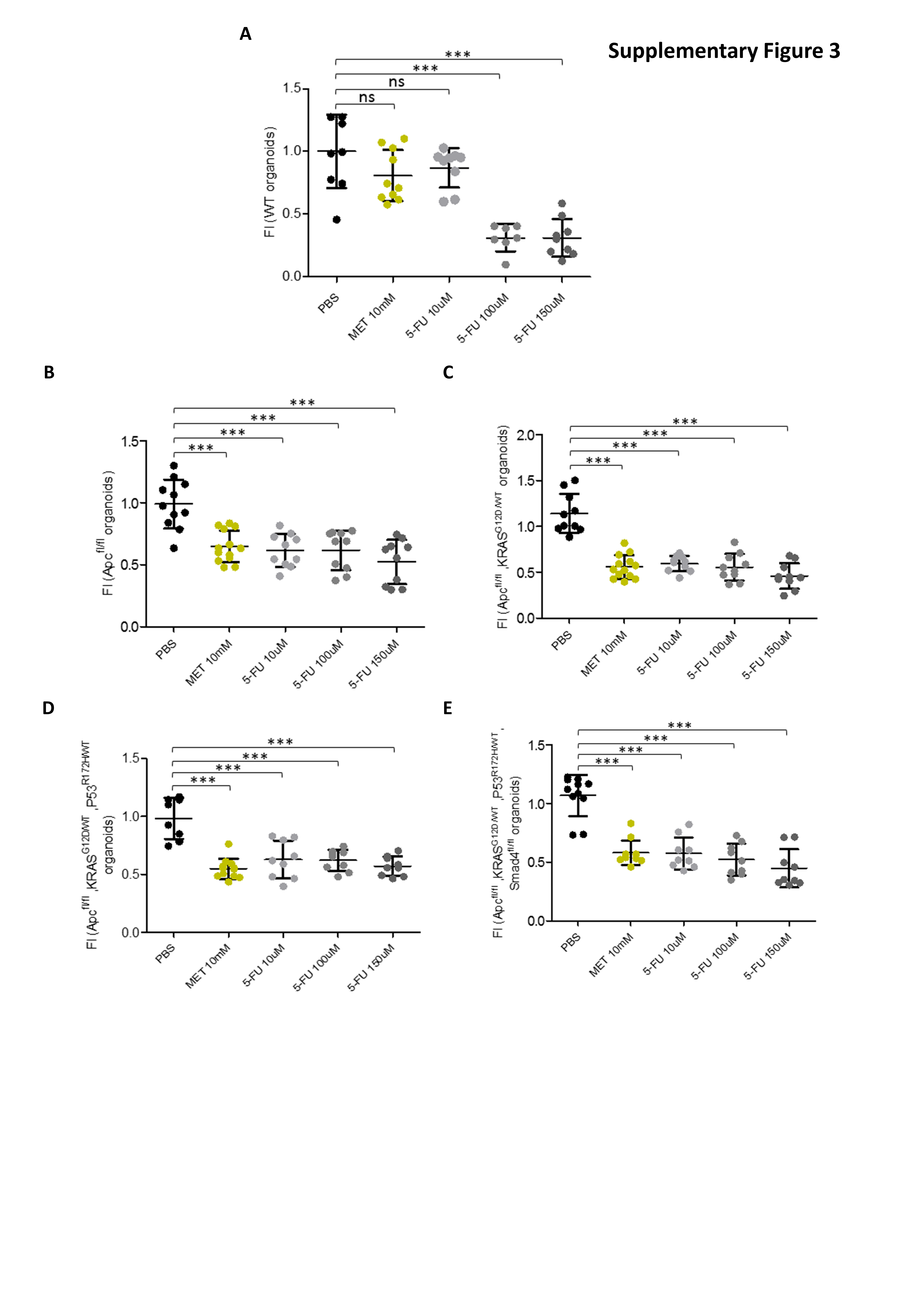

Supplement: S3 Fig — MTT cell viability assays upon 48 hours treatments with PBS (black bars), 10 mM metformin (yellow bars) or 10, 100 and 150 uM 5-FU (grey bars) in the different CRC-like organoids representative stages: WT organoids (A); APCfl/fl organoids resembling stage I (B); APCfl/fl, KRASG12D/WT organoids resembling stage II (C); APCfl/fl, KRASG12D/WT, P53R172H/WT organoids resembling stage III (D); APCfl/fl, KRASG12D/WT, P53R172H/WT, Smad4fl/fl organoids resembling stage IV (E). Data are represented by the fold-change mean ±SD (n = 3) in all the plots. (ns, P > 0.05; *, P ≤ 0.05; **, P ≤ 0.01; ***, P ≤ 0.001). (TIF) [file pone.0219944.s003.TIF]

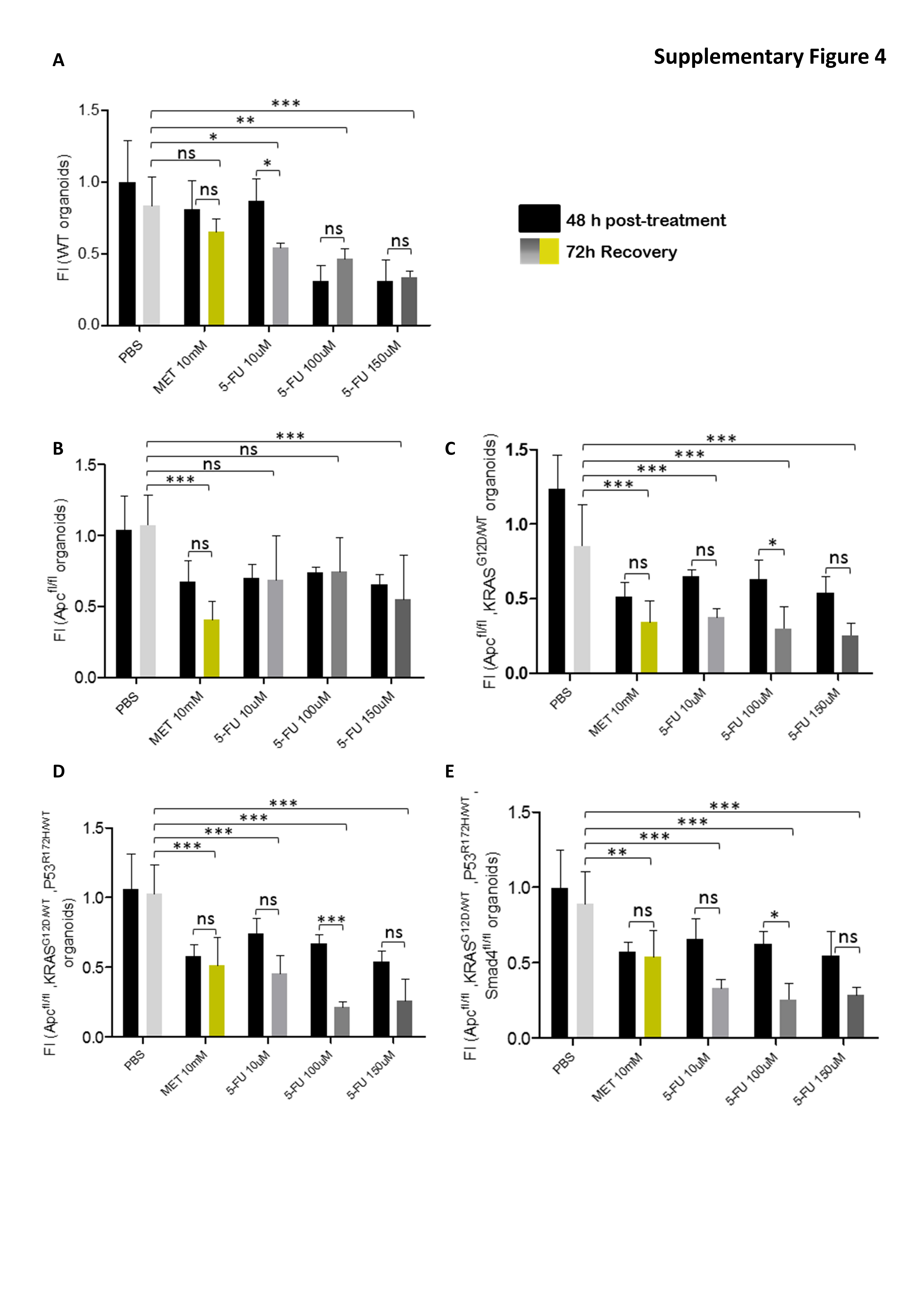

Supplement: S4 Fig — MTT cell viability assays upon 48 hours treatments (black bars) and upon extra 72h post-treatment recovery with PBS (light grey bars), 10 mM metformin (yellow bars) or 10, 100 and 150 uM 5-FU (dark grey bars) in the different CRC-like organoids representative stages: WT organoids (A); APCfl/fl organoids resembling stage I (B); APCfl/fl, KRASG12D/WT organoids resembling stage II (C); APCfl/fl, KRASG12D/WT, P53fl/R172H organoids resembling stage III (D); APCfl/fl, KRASG12D/WT, P53fl/R172H,Smad4fl/fl organoids resembling stage IV (E). Data are represented by the fold-change mean ±SD (n = 3) in all the plots. (ns, P > 0.05; *, P ≤ 0.05; **, P ≤ 0.01; ***, P ≤ 0.001). (TIF) [file pone.0219944.s004.TIF]

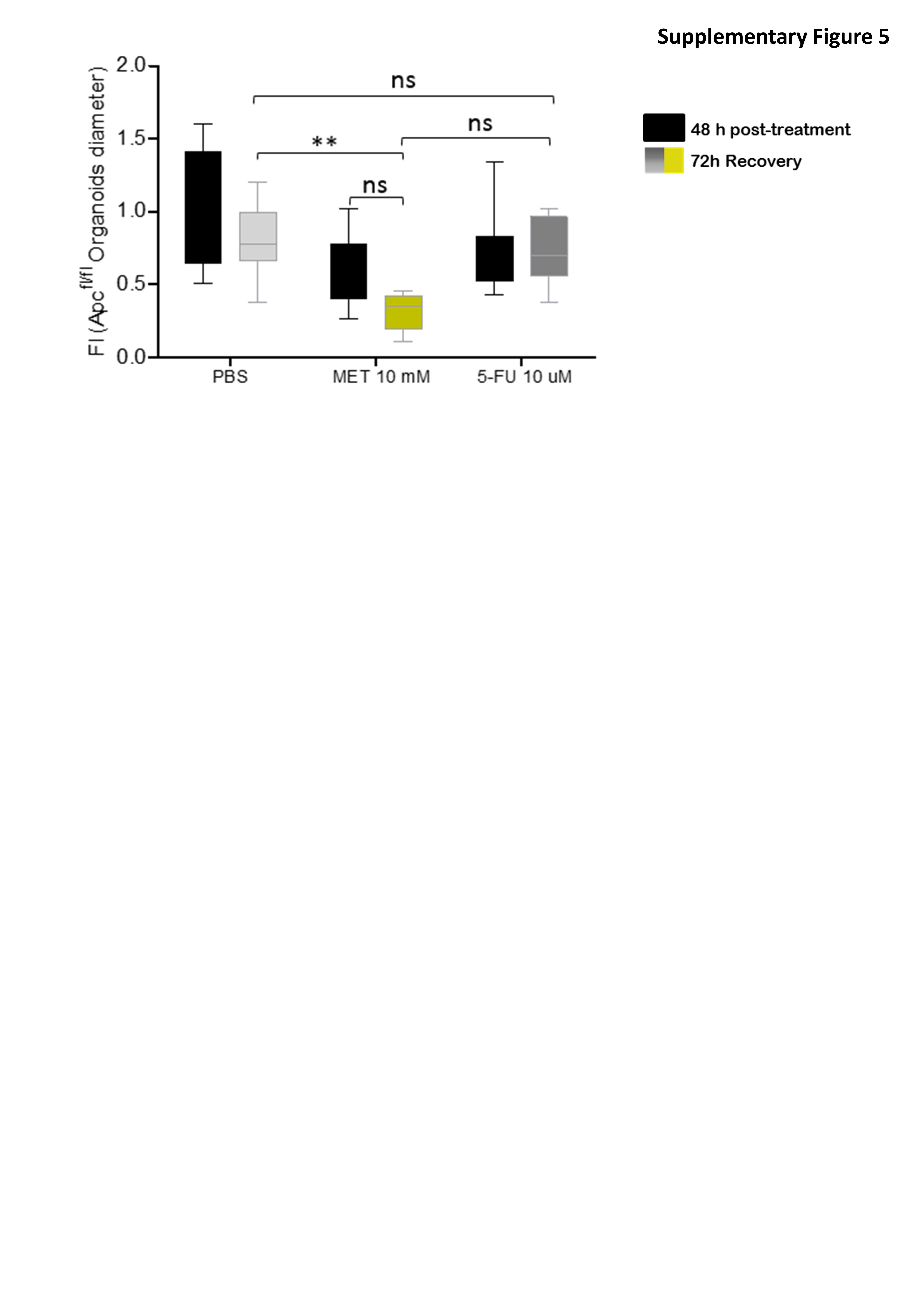

Supplement: S5 Fig — Organoids diameter measured with Image J 1.48 program from pictures of the MTT cell viability assays upon 48 hours treatments (black bars) and upon extra 72h post-treatment recovery with PBS (light grey bars), 10 mM metformin (yellow bars) or 10 μM 5-FU (dark grey bars) in APCfl/fl organoids resembling stage I. Data are represented by the fold-change mean ±SD (n = 3 experiments/3 images analyzed per condition) (ns, P> 0.05; *, P ≤ 0.05; **, P ≤ 0.01). Significance between groups was determined by unpaired t-test analyses. Pictures were captured using the x10 objective, in bright field. Leica microscope (Leica Microsystems). (TIF) [file pone.0219944.s005.TIF]

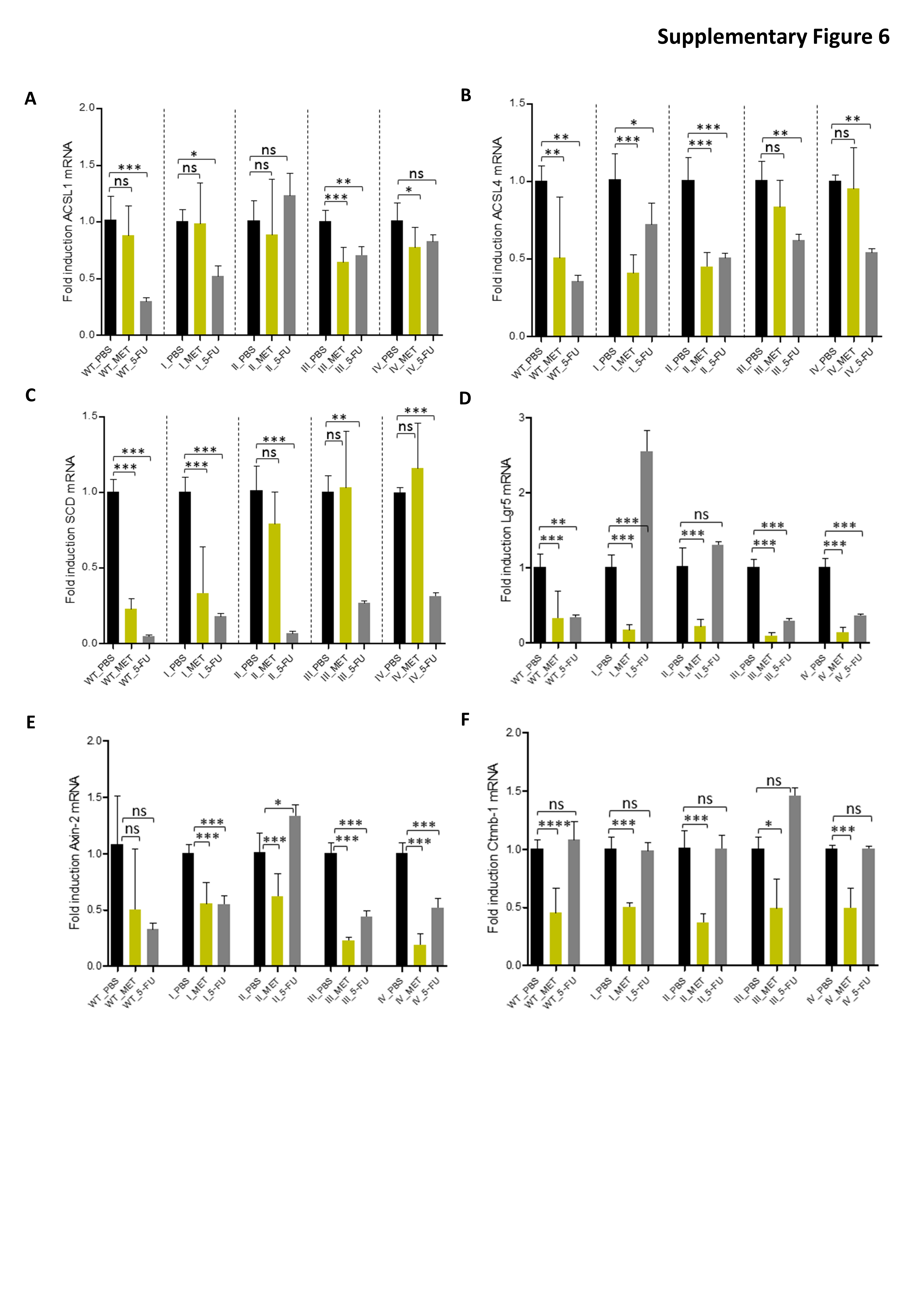

Supplement: S6 Fig — Expression levels of enzymes related to the ACSL/SCD axis, ACSL1 (A), ACSL4 (B) and SCD (C) by RT-QPCR; and expression levels of different stem cell markers, Lgr5 (D), Axin-2 (E) and Ctnnb-1 (F) by RT-QPCR; upon PBS (black bars), 10 mM metformin (yellow bars) or 10, 100 and 150 uM 5-FU (grey bars). Data are represented by the fold-change mean ±SD (n = 3). (ns, P> 0.05; *, P ≤ 0.05; **, P ≤ 0.01; ***, P ≤ 0.001). (TIF) [file pone.0219944.s006.TIF]

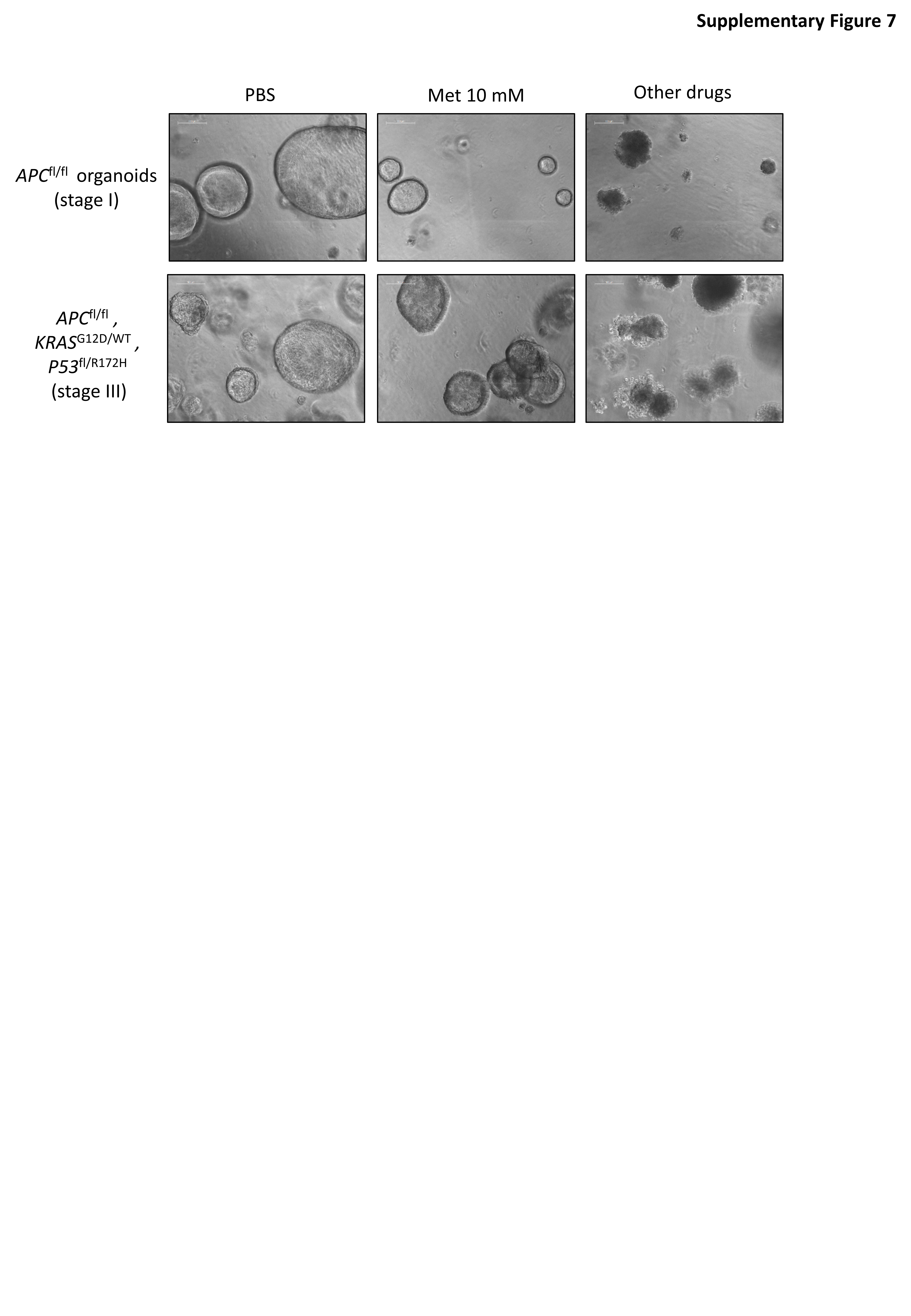

Supplement: S7 Fig — Organoids (stage I and III) representative pictures with DMSO, metformin and other metabolic drugs against CRC progression, upon 48 hours treatments plus upon extra 72h post-treatment recovery. Pictures were captured using the × 10 objective, in bright field. Leica microscope (Leica Microsystems). (TIF) [file pone.0219944.s007.TIF]
