# Supplementary material for: A more physiological approach to lipid metabolism alterations in cancer: CRC-like organoids assessment
Source: PLoS One. 2019 Jul 24;14(7):e0219944. doi: 10.1371/journal.pone.0219944 (PMC6655698; doi:10.1371/journal.pone.0219944)
Supplement: S1 Table — (DOCX) [file pone.0219944.s008.docx]

| **ACSL1** | TGACCTCTCCATGCAGTCAG/ AGCCTATGCACTCAGCCAGT |
| --- | --- |
| **ACSL4** | CACCATTGCCATTTTCTGTG/ GCCTTCAGTTTGCTTTCCAG |
| **SCD** | TTCTTACACGACCACCACCA/ GCAGGAGGGAACCAGTATGA |
| **CTNNB1** | TAACTATCAGGATGACGCGG/ TTAACTACCACCTGGTCCTC |
| **AXIN-2** | GGACTGGGGAGCCTAAAGGT/AAGGAGGGACTCCATCTACGC |
| **LGR5** | GGACCAGATGCGATACCGC/ CAGAGGCGATGTAGGAGACTG |
| **B2M** | AGACTGATACATACGCCTGC/ ATCACATGTCTCGATCCCAG |

**S1 Table**: Primers´ sequences (Invivogen) used for quantitative real-time PCR.
