# Supplementary material for: A more physiological approach to lipid metabolism alterations in cancer: CRC-like organoids assessment
Source: PLoS One. 2019 Jul 24;14(7):e0219944. doi: 10.1371/journal.pone.0219944 (PMC6655698; doi:10.1371/journal.pone.0219944)
Supplement: S2 Table — (DOCX) [file pone.0219944.s009.docx]

**S2 Table**: Probes from TaqMan® MicroRNA Assays (ThermoFisher) used for quantitative real-time PCR.

miRBase ID Cat No

| mmu-miR-19b-3p | 4427975 |
| --- | --- |
| mmu-miR-19b-1-5p | 4427975 |
| mmu-miR-142a-3p | 4427975 |
| mmu-miR-142a-5p | 4427975 |
| U6 snRNA | 4427975 |
